# A Copula Entropy Approach to Dependence Measurement for Multiple Degradation Processes

**DOI:** 10.3390/e21080724

**Published:** 2019-07-25

**Authors:** Fuqiang Sun, Wendi Zhang, Ning Wang, Wei Zhang

**Affiliations:** 1 Science and Technology on Reliability and Environmental Engineering Laboratory, School of Reliability and Systems Engineering, Beihang University, Beijing 100191, China; 2 Beijing Aeronautical Science & Technology Research Institute, Commercial Aircraft Corporation of China Ltd., Beijing 102211, China

**Keywords:** copula entropy, measure, dependence, multiple degradation processes

## Abstract

Degradation analysis has been widely used in reliability modeling problems of complex systems. A system with complex structure and various functions may have multiple degradation features, and any of them may be a cause of product failure. Typically, these features are not independent of each other, and the dependence of multiple degradation processes in a system cannot be ignored. Therefore, the premise of multivariate degradation modeling is to capture and measure the dependence among multiple features. To address this problem, this paper adopts copula entropy, which is a combination of the copula function and information entropy theory, to measure the dependence among different degradation processes. The copula function was employed to identify the complex dependence structure of performance features, and information entropy theory was used to quantify the degree of dependence. An engineering case was utilized to illustrate the effectiveness of the proposed method. The results show that this method is valid for the dependence measurement of multiple degradation processes.

## 1. Introduction

Degradation is seemingly fundamental to all things in nature [1]. Therefore, the failure mechanism of a highly reliable system usually can be traced to underlying degradation processes such as the fatigue and corrosion of metal materials, the wear of mechanical parts, the parametric drift of semiconductor devices, and other processes [2]. As a consequence, degradation modeling has become an efficient method to evaluate the reliability of long lifetime products, combining the probabilistic degradation process and the fixed failure threshold [3].

Modern engineering systems may have multiple degradation features because of their complex structures and functions [4], and any of them that exceed the defined threshold may cause product failure [5,6]. Since all degradation features of a product share several common factors (e.g., the same inside structures, user experience, environmental/operational conditions, and maintenance history), it is unavoidable that there is dependence among multiple degradation features. This dependence structure may be linear or nonlinear. When ignoring the existence of dependence among multiple degradation features, degradation modeling and lifetime estimation under the premise of the independence assumption may lead to less credible or erroneous results. Therefore, it is safer to describe and measure dependence accurately and reasonably among multiple degradation features prior to modeling.

The associated relationships of multiple random variables are usually described by correlation and dependence. The differences and relationships between dependence and correlation are elaborated as following firstly.

The correlation is usually utilized to describe linear relationships. It does not certainly mean that X and Y are independent of each other when there is no correlation between X and Y. The Pearson correlation coefficient, based on the multivariate normality assumption, is often employed to measure the linear relationships between two random variables in statistics [7]. Xu et al. [8] adopted the Pearson correlation coefficient to calculate the correlation between two degradation processes. However, the Pearson correlation coefficient can only be used to measure linear relationships. For example, the random variable X follows the standard normal distribution and the random variable Y = X^2^. Obviously, there is a strong dependence between X and Y, and the value of Y can be completely determined by X. However, the correlation coefficient between them is 0. Therefore, the Pearson correlation coefficient has some shortcomings in measuring the associated relationships of random variables [9]. It will misestimate the dependence between two variables when the sample size is not large enough or the dependence relationship is nonlinear [10].

Dependence is the opposite of independence, which means that the random variables X and Y have no independence in probability characteristics. Dependence usually contains both linear and nonlinear relationships. Therefore, it is more appropriate to use dependence to describe the relationship between random variables [11]. Dependence measurement is how the dependence between variables or the dependence between distribution functions of variables is measured [12]. The traditional modeling method based on multidimensional joint distribution relies on the correlation coefficient. For two-dimensional normal random variables (X, Y), the correlation coefficient of X and Y is 0 means that X and Y are independent of each other, and it is not applicable to nonlinear relationships.

The rank correlation coefficient can be utilized to estimate the nonlinear dependence relationship between two variables, and it has no restriction regarding the distribution of variables. The rank correlation coefficient primarily includes the Kendall correlation coefficient and the Spearman correlation coefficient [13,14], and their original purpose was to measure and estimate dependence in the psychiatric symptom rating field. Nelsen [12] adopted the link function between the copulas and Kendall’s *τ* (or Spearman’s *ρ*) to assess the dependence of bivariate degradation data. Similarly, Wang and Pham [5], Sari et al. [15], and Sun et al. [16] also adopted the rank correlation coefficient and copulas to measure the dependence between two performance characters. One major disadvantage of the rank correlation coefficient is that there is a loss of information when the data are converted to ranks [17]. Furthermore, they cannot be used to detect dependence when more than two variables are involved [10]. In a multivariate context, in general, it is more important to study multivariate association than a bivariate association.

Therefore, it is difficult to use the existing methods to accurately measure the dynamic and nonlinear characteristics of dependence measurements of multiple degradation processes. Indeed, it is necessary to find a more suitable measurement method to calculate the dependence among multiple degradation features of a product. Schmid et al. [18] proposed a method of multivariate association measurement based on copula, which extended the commonly used bivariate measurement method to multivariate and applied copula to measure multivariate association. Ane et al. [19] applied copula to the financial area and proposed a measurement method of association between the financial risks based on copula. As a more useful alternative, the copula entropy, which combines information entropy and the copula theory, is proposed to measure dependence among multiple variables. Copula entropy can measure association information and dependence structure information simultaneously. Moreover, copula entropy does not impose constraints on the dimension of multiple variables. Due to these advantages, copula entropy has attracted much interest for its ability to measure multivariate dependence in many fields, and copula entropy has been gradually applied in hydrology, finance and other fields. Singh and Zhang [20] discussed the flexibility to model nonlinear dependence structure using parametric copulas (e.g., Archimedean, extreme value, meta-elliptical, etc.) with respect to multivariate modeling in water engineering. Zhao et al. [21] used the copula entropy model to measure the stock market correlations, compared with the linear correlation coefficient and mutual information methods, which have the advantages of dimensionless, and able to capture non-linear correlations. Hao et al. [22] introduced the integration of entropy and copula theories to the hydrologic modeling and analysis area. Chen et al. [10,23] used the copula entropy to computed the dependence between the mainstream and its upper tributaries and also used the copula entropy coupled with an artificial neural network to calculate the correlation between each input and output of the neural network for rainfall-runoff simulation. Ma and Sun [24] proved the equivalence between copula entropy and mutual information, and mutual information is essentially an entropy. Xu et al. [25] proposed the copula theory to quantitatively describe the connection of bivariate variables or multivariate variables in the hydrometeorological field. Similarly, Huang et al. [26] applied copula entropy to measure dependencies between traffic noise and traffic flow. Salimi et al. [27] used copula entropy to capture the dependencies among the sub-components of the system in the modeling of complex service systems.

In this paper, a novel measurement method that uses copula entropy is proposed to measure the dependence among multiple degradation features. First, the copula function and information entropy theory were employed to build the copula entropy. The former was used to describe the dependence structure among variables, and the latter was utilized to quantify the dependence. Then, the copula entropy of multiple degradation processes was calculated. Parameter estimation of copula entropy was performed using the maximum likelihood estimate (MLE) method. The Akaike information criterion (AIC) was adopted to select the most suitable copulas. Finally, a case study with multivariable degradation data of a microwave electronic assembly was studied to validate the proposed method. The proposed copula entropy method could address two problems in the dependent measurement of multiple degradation processes: the first is how to measure and directly compare the dependence between every two pairs of the degradation processes, and the other is how to measure directly compare the dependence among multiple degradation processes at different phases.

The paper is organized as follows. Section 2 presents the theory of the copula function and information entropy. This section also combines these to build the copula entropy theory. Section 3 elaborates on the calculation methods of copula entropy, including the calculation of the cumulative distribution function, the method of parameter estimation, and the Monte Carlo simulation calculation. Section 4 provides the case study, and Section 5 concludes the paper.

## 2. Copula Entropy Theory

### 2.1. Multivariate Copula Function

It is difficult to identify the multivariate probability distribution because of the complexity and the high dimension of marginal distributions. The copulas separate the learning of the marginal distributions from the learning of the multivariate dependence structure to simplify this process [28].

The below theorem provides the necessary and sufficient conditions for copula theory. It explains the effect of copulas in expressing the relationship between the multivariate distribution and the relevant univariate marginal distributions [6].

**Theorem** **1.** *(Sklar’s theorem [29]): Let X = (x_1_, x_2_,…, x_n_) be a random variable, and its marginal distributions are F_1_(x_1_), F_2_(x_2_),…, F_n_(x_n_), and H is their joint distribution function. Then, the copula function C is presented such that*

(1) $$H\left(x_{1},x_{2},\ldots ,x_{n}\right)=C\left(F_{1}\left(x_{1}\right),F_{2}\left(x_{2}\right),\cdots ,F_{n}\left(x_{n}\right)\right)$$

The copula *C* is unique when *F*_1_(*x*_1_), *F*_2_(*x*_2_), …, *F_n_*(*x_n_*) are continuous. On the contrary, the function *H*, defined by Equation (1), will be the joint distribution function of the margins *F*_1_(*x*_1_), *F*_2_(*x*_2_), …, *F_n_*(*x_n_*) if *F*_1_(*x*_1_), *F*_2_(*x*_2_), …, *F_n_*(*x_n_*) are univariate distributions.

The multivariate copula function can be defined according to the theorem.

**Definition** **1.** (n-dimensional copula) [12]: An n-dimensional copula is a function C from I^n^ = [0, 1]^n^ to I and it must have the following properties:

*(1)*  *If u* = (*u*_1_, …, *u_n_*) = 1, *then* *C*(*u*) *= 1;*
*(2)*  *For everyu* = (*u*_1_, …, *u_n_*) in *I^n^*, *if at least one coordinate of* *u* *is 0 then* *C*(*u*) *= 0;*
*(3)*  *If all coordinates of u except u_k_ are 1, then*
  (2) $$C(u)=C\left(1,\ldots ,1;u_{k};1,\ldots ,1\right)=u_{k}$$
*(4)*  *For each hyper rectangle*$B=\prod_{i=1}^{n}\left[u_{i},v_{i}\right]\subseteq {\left[0,1\right]}^{n}$*, theC-volume ofBis non-negative*
  (3) $$\int_{B}^{}dC([u,v])=\sum_{z\in \times \underset{i=1}{n}\{u_{i},v_{i}\}}{\left(-1\right)}^{n(z)}C(z)\geq 0$$
  *wheren*(*z*) = #{*k*:*z_k_* = *u_k_*}.

The density of a copula function *C* is denoted by *c*, which may be achieved by taking the partial derivatives as

(4) $$c\left(u_{1},u_{2},\ldots ,u_{n}\right)=\frac{\partial^{n}c\left(u_{1},u_{2},\ldots ,u_{n}\right)}{\partial u_{1}\partial u_{2}\ldots \partial u_{n}}\forall u=(u_{1},u_{2},\ldots ,u_{n})\in I^{n}$$

Based on multivariate differentiation, the joint density function corresponding to the distribution function, *H*(*u*_1_, *u*_2_, …, *u_n_*), can be calculated by

(5) $$h\left(u_{1},u_{2},\ldots ,u_{n}\right)=c\left(F_{1}\left(x_{1}\right),F_{2}\left(x_{2}\right),\cdots ,F_{n}\left(x_{n}\right)\right)f_{1}\left(x_{1}\right)f_{2}\left(x_{2}\right)\cdots f_{n}\left(x_{n}\right)$$

where *u*_1_ = *F*_1_(*x*_1_), *u*_2_ = *F*_2_(*x*_2_), …, *u_n_* = *F_n_*(*x_n_*) and *f*_1_(*x*_1_), *f*_2_(*x*_2_), …, *f_n_*(*x_n_*) are the probability density functions of marginal distribution function *F*_1_(*x*_1_), *F*_2_(*x*_2_), …, *F_n_*(*x_n_*), respectively.

Copula functions have many types, and different types can reflect different dependence structures. Table 1 shows a few typical copula functions.

### 2.2. Information Entropy

The entropy originated from the thermodynamics first and then gradually extended to the study of information theory. It is called information entropy in the information field and measures the uncertainty of information. Shannon [30] first proposed the concept of information entropy as follows:

(1) The function, *S,* is continuous and the probability is *p_i_*;
(2) Under the condition of equivalence probability, *S* is a monotonically increasing function with the possible result quantity *n*;
(3) For two mutually independent events in *S*, the uncertainty between them is the sum of the uncertainties when considering them separately.

Then *S* can be named as the information entropy function.

Let X be a random variable with a probability *p_i_*, the entropy of X is given by [31]:

(6) $$S(x)=-k\sum_{i=1}^{n}p_{i}\log p_{i}$$

Information entropy has the following properties:

(1) *S_n_*(*p*_1_, *p*_2_, …, *p_n_*) ≥ 0;
(2) If *p_k_* = 1, then *S_n_*(*p*_1_, *p*_2_, …, *p_n_*) = 0, where *Sn*(0, ..., 0, 1, 0, ..., 0) = 0;
(3) *S_n+_*_1_(*p*_1_, *p*_2_, …, *p_n_, p_n+_*_1_ = 0) = *S_n_*(*p*_1_, *p*_2_, …, *p_n_*);
(4) *S_n_*(*p*_1_, *p*_2_, …, *p_n_*) ≤ *S_n_*(1/*n*, 1/*n*, …, 1/*n*) = ln(*n*);
(5) *S_n_*(*p*_1_, *p*_2_, …, *p_n_*) is a symmetric concave function on all variables.

where $S\left(p_{1},p_{2},\ldots ,p_{n}\right)=-\sum_{i=1}^{n}p_{i}\log p_{i}$.

Information entropy gives a quantitative measurement of the degree of uncertainty in the information. From its calculation formula, it can be seen that the probability distribution of *p_i_* needs to be determined to carry out the calculation. However, the probability, *p_i_*, in each case cannot be actually determined in practical calculations. Since the distribution of information cannot be directly obtained in many cases, only the average, variance, and other parameters of the distribution dependence information can be obtained through experiments.

In the case of an unknown distribution, the distribution needs to be determined according to the known distribution dependence information. Therefore, the final distribution must be a distribution that corresponds to the maximum entropy function under the premise of satisfying all known information. A maximum entropy method of estimation has been proposed by Behrouz [32] that is used to derive the minimum bias probability distribution for the given information based on constraints. It can be expressed as

(7) $$\max S(x)\;s.t.\;E(g_{j}(x))=c_{i},j=1,2\ldots m,$$

where *S*(*x*) is given in Equation (6), *g_j_*(*x*) is a feature function, and *c_j_* is the expected value of the *j*-th feature.

### 2.3. The Selection of Copulas

For the application of the multivariate copulas, an important question is how to select the most suitable copula from a set of given candidate copulas to describe the dependence structure.

One commonly used method is the Akaike information criterion (AIC). The Akaike information criterion [33] is a standard used to measure the goodness of statistical model fitting. It is based on the concept of entropy, and it can weigh the complexity of an estimated model and the goodness of the model-fit data. AIC is defined as follows:

(8) AIC=−2ln(L)+2k,

where *k* is the number of parameters in the model; *L* is the likelihood function value. The smaller the value of AIC is, the fitter the dependence structure is.

Another commonly used criterion is the Bayesian Information Criterions (BIC) [34], which is defined as

(9) BIC=−2ln(L)+k⋅ln(n),

where *n* is the sample size. Similar to AIC, the smaller the BIC value is, and the better the fitting degree of the model is.

In addition, the likelihood function could be also used to select copulas. The essence is to compare the maximum value of the likelihood function under the constraint condition with the maximum value of the likelihood function without the constraint condition [35]. The larger the maximum value of the likelihood function is, the better the model fitting degree is.

### 2.4. Copula Entropy

#### 2.4.1. Definition of Copula Entropy

James and Crutcheld [36] demonstrate that Shannon information measures can fail to accurately ascertain multivariate dependencies due to the conflation of different relationships among variables. Thus, we chose the copula entropy, which combines the information entropy and the copula function, to describe the dependence relationship of multivariate.

Copula entropy is a combination of copula theory and maximum entropy theory. The copula function is used to describe the dependence among variables, and information entropy theory is utilized to quantify the dependence. The entropy variables are mutually independent in the entropy model, which is a general assumption for the principle of maximum entropy [37]. However, the copula theory needs to be supported to describe the entropy variable with dependence. Based on the copula theory of Sklar [29], joint entropy can be expressed as the sum of *n* univariate entropy and copula entropy. From this, the functional form [21] of the copula entropy used in this paper is

(10) $$Hc(u_{1},u_{2},\cdots ,u_{d})=-\int_{0}^{1}\cdots \int_{0}^{1}c(u_{1},u_{2},\cdots ,u_{d})\ln(c(u_{1},u_{2},\cdots ,u_{d}))du_{1},\cdots ,du_{d},$$

where *c* (*u*_1_, *u*_2_, ..., *u_d_*) is the probability density function of the copula function; *u_i_* = *F_i_* (*x_i_*) = *P* (*x_i_* ≤ *X_i_*), *i* = 1, 2, ..., *d*, represents the marginal distribution function of random variables.

(1) The characteristics of copula entropy can be deduced based on the three properties of the entropy function [21,38], copula entropy should be continuous [39].
(2) If all the discrete probabilities of the copula are equal, then it should be a monotonically increasing function [40], and the measurement of uncertainty should be higher when there are more possible outcomes than when there are few.
(3) The monotonicity property of copula entropy can be deduced from that the copula function is monotonic [12].

Take Gumbel copula, for example, the mathematical expression for its copula entropy is given as below.

(11) $$Hc(u_{1},u_{2},\cdots ,u_{d})=-\int_{0}^{1}\cdots \int_{0}^{1}\exp\left\{-{\left[\sum_{i=1}^{d}{\left(-\ln u_{i}\right)}^{\theta }\right]}^{1/\theta }\right\}\ln(\exp\left\{-{\left[\sum_{i=1}^{d}{\left(-\ln u_{i}\right)}^{\theta }\right]}^{1/\theta }\right\})du_{1},\cdots ,du_{d}$$

As shown in Equation (11), the Gumbel function is monotonic and therefore its copula entropy is monotonic.

Copula entropy is dimensioned as entropy, and its unit of measurement is the nat [21]. The nat is the natural unit of information. Sometimes nit or nepit is also used as the unit of information or entropy and is based on natural logarithms and powers of *e*, rather than the powers of 2 and base 2 logarithms, which define the bit. It can be expressed as the following equation:

(12) $$2^{x}=e^{1}\Rightarrow x=\frac{1}{\ln2},$$

where *x* stands for one nat; and *e* is the base of the natural logarithm.

This unit is also known by its unit symbol, the nat. The nat is the coherent unit of information entropy. The International System of Units, by assigning the same units (joule per kelvin) both to heat capacity and to thermodynamic entropy, implicitly treats information entropy as a quantity of dimension one, with 1 nat = 1. Physical systems of natural units that normalize Boltzmann’s constant to 1 effectively measure the thermodynamic entropy in nats. When the Shannon entropy is written using a natural logarithm, as in Equation (12), it is giving a value measured in nats.

According to information entropy theory, when the known information decreases, the corresponding entropy value becomes larger. In contrast, when the known information becomes larger, the corresponding entropy value becomes smaller. When the above properties are applied to copula entropies, the lower the dependence degree among the variables, the weaker the corresponding dependence information, and the larger the entropy value reflected in copula entropy. Similarly, the higher the dependence among variables, the stronger the corresponding dependence information, and the smaller the entropy of copula entropy. Copula entropy can be calculated using multidimensional integration, and its value range is a real number space.

#### 2.4.2. Copula Entropy and the Pearson Correlation Coefficient

Copula entropy, as a newly developed measurement of dependence, has some advantages not found in other dependence measurements. As the copula function can describe nonlinear dependence, copula entropy can also measure the information of a nonlinear dependence structure. In addition, it is possible for copula entropy to obtain unitary results to achieve a direct comparison since entropy has a dimension. Therefore, copula entropy can measure the dependence of two or more variables.

Dependence among variables has been widely studied. The traditional dependence measurement method is based on the correlation coefficient. Although this method is currently widely used for dependence measurement, the correlation coefficient has some obvious limitations. In contrast, copula entropy theory is applied to nonlinear correlation modeling in this paper instead of relying on correlation coefficients [41]. In addition, a comparison of the copula entropy method and the correlation coefficient is given in Table 2.

By comparing the information in the table, the following conclusions can be made. First, the correlation coefficient method only applies to the linear correlation. However, in practice, the relationship among the variables is not always an ideal linear relationship. However, nonlinear dependence is quite natural in many complex engineering applications; in this respect, copula entropy can be used to measure both linear and nonlinear correlation to solve dynamic nonlinear correlation measurement problems for multiple degradation processes.

Second, the correlation coefficient method often focuses on the degree of dependence. But another important aspect of this relationship is the structure of dependence, which is often omitted and ignored [42]. However, copula entropy focuses on not only the degree of dependence but also the structure of dependence. The copula entropy method can more accurately describe the relationship among variables.

Third, the correlation coefficient method is dimensionless, and it is difficult to compare in cases with more than two sets of variables. However, copula entropy has dimension and can be compared directly, and a comparison of the dependence among multiple variables can be obtained. For example, if the correlation coefficient between “A” and “B” is 0.6, and the correlation coefficient between “B” and “C” is 0.3, then one can conclude that “AB” is more correlated than “BC.” However, if the correlation coefficient between “A” and “B” is 0.6, and the coefficient between “C” and “D” is 0.3, then the correlation between “AB” and “CD” cannot be compared. In contrast, copula entropy is comparable and is easily explained with entropy theory. In information theory, entropy has its own unit, the nat, which is used to measure the information obtained from variables. Therefore, if the value of the copula entropy between “A” and “B” is less than that between “C” and “D,” this means that the dependence of “AB” is higher than that of “CD.”

In summary, copula entropy can accomplish the following two issues that traditional dependence measurement methods cannot achieve. The nonlinear dependence will be measured using the copula method, and it can be used to analyze nonlinear dependence among variables, instead of just focusing on linear dependence among variables. The dependence between any two degradation processes can be directly compared without intermediate variables. Therefore, the dependence among three or more variables can be compared. In addition, it is possible to compare the dependencies of variables during different time phases and determine the time-varying law of dependence.

## 3. Dependence Measurement of Multivariate Degradation Processes

### 3.1. Problem Description

Some products with complex system structures, various performance features, and varying operational conditions tend to exhibit degradation of multiple performance features, and there are unavoidable dependencies among these degradation features. The dependencies among the degradation processes of multiple performance features often show dynamic and nonlinear statistical features. If these dependencies are ignored, product degradation modeling and lifetime estimation may result in less credible or even erroneous results.

Some products possess a simple degradation mechanism, and the reliability of the product can be directly derived by using the relationship among the degradation features amount and time (i.e., the product performance degradation trajectory). However, some products possess complex degradation mechanisms, and the quantitative relationship of the degradation model cannot be expressed directly. In this case, traditional dependence measurement methods cannot accurately obtain dependence information. This results in the inability to measure dependence or to obtain accurate results.

As mentioned above, simple linear correlation coefficients cover a wide range of values that can reveal a variety of dependency relationships. However, the information of the nonlinear relationships is ignored if they are not simply linearly correlated. The traditional dependence measurement method is only applicable to linear correlations and can only measure the dependence between two variables. The copula entropy proposed in this paper can be applied to correct this ignorance, and it can measure nonlinear dependence relationships among two or more variables. In addition, copula entropy has no dimensional constraints; hence, enough indicators can be chosen to measure dependence among the variables.

### 3.2. The Calculation Process

**Figure 1:**
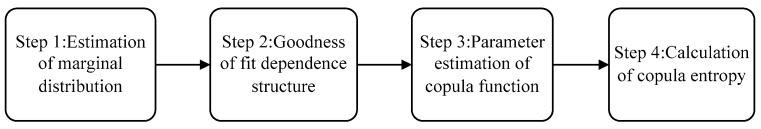
Dependence measurement of the copula entropy method flowchart.

The essence of copula entropy is a multivariate integral that can be calculated using the integral method. However, when the dimension of the integrand is high and the form is complex, the calculation process will be very difficult. To this end, the Monte Carlo simulation method would be used to calculate the copula entropy. The method is divided into four steps, as shown in Figure 1.

The specific steps are described as follows:

- Step 1: The kernel density estimation method is used to estimate the marginal distribution and calculate the cumulative distribution function (CDF) of every performance feature degradation increasement.
- Step 2: A different type of copula function is adopted to combine the cumulative distribution functions of different performance feature degradation increasement separately. In addition, the Akaike information criterion (AIC) is performed to compare the goodness-of-fit of the copula function.
- Step 3: Parameter estimation of the copula function is performed using the maximum likelihood estimate (MLE) method. Also, the establishment of the copula entropy function is performed based on the chosen copula function and the determination of integrand function.
- Step 4: The Monte Carlo simulation method is utilized to calculate the copula entropy.

#### 3.2.1. Estimation of Marginal Distribution

In the copula entropy method, the structure of the marginal distribution is an important issue. The first step is to estimate the marginal distribution. For degradation data of multi-performance features, the cumulative distribution function of the degradation increments for each performance feature needs to be calculated. In this step, the kernel density estimation method [43] is applied to estimate and calculate the cumulative distribution function of degradation increasement.

Kernel density estimation is used in probability theory to estimate unknown density functions. It is also one of the non-parametric test methods proposed by Rosenblatt [44] and Parzen [45]. Based on the univariate kernel density estimation, a risk value prediction model can be established. Different risk value prediction models can be established by weighting the variation coefficient of kernel density estimation. Since the kernel density estimation method does not use prior knowledge of the data distribution and does not attach any assumptions to the data distribution, it is a method for studying the data distribution features from the data sample itself. Therefore, it has received great attention in the field of statistical theory and application.

For a certain performance feature, the degradation data are *X*_1_, *X*_2_, …, *X_m_*, then the degradation increment is

(13) $$\Delta X_{i}=X_{i}-X_{i-1},$$

where *i* is the data coefficient, and *i* = 1, 2, …, *m*, *m* represents the number of data observations.

The degradation increment of each performance feature is assumed to confirm the basic requirements of the statistical test used, such as independence and distribution. Then, the probability density function, *p_i_*(*x*), of the *i*th performance feature degradation increment is calculated as [46]

(14) $$p_{i}\left(\Delta x\right)=\frac{1}{mh}\sum_{t=1}^{T}K\left(\frac{\Delta x-\Delta X_{t}}{h}\right)$$

(15) $$K\left(\frac{\Delta x-\Delta X_{t}}{h}\right)=\frac{1}{\sqrt{2\pi }}\exp\left(-\frac{{\left(\Delta x-\Delta X\right)}^{2}}{2h^{2}}\right),.$$

where *t* is the time interval; *T* is the width of the time interval; *h* is the width of the form smooth parameter; and *K*(·) is a kernel function, which is a standard Gaussian distribution [47] with expectation 0 variance of 1, *i* = 1, 2, …, *d*; and *d* is the number of performance features.

Therefore, the cumulative distribution function, *u_i_*, of the *i*-th performance feature degradation increment is

(16) $$u_{i}=\int p_{i}\left(\Delta x\right)d\Delta x.$$

#### 3.2.2. Goodness of Fit Dependence Structure

As mentioned in Section 2.3, there are three commonly used methods for the selection of copulas, including AIC, BIC and the likelihood function method. Generally speaking, AIC is the most commonly used method in choosing copula. The difference between AIC and BIC mainly lies in the number of model parameters.

As the number of unknown parameters of the candidate copulas is the same [12], the more commonly used AIC method is chosen. Similarly, for the likelihood function method, the larger the likelihood function is, the smaller the AIC value is, and the fitter the dependence structure is. Therefore, ultimately the AIC method is chosen for the goodness of fit in the dependence structure.

#### 3.2.3. Parameter Estimation of Copula Entropy

After the copula entropy is built, its internal parameters need to be estimated. The maximum likelihood estimation method (MLE) is the method used to solve this problem. Suppose the probability distribution function of variable *x_i_* is expressed as *F_i_*(*x_i_*; *φ_i_*), *i* = 1, 2, …, *n*, the probability density function is *f_i_*(*x_i_*; *φ_i_*), and *φ_i_* is the unknown parameter in each function. Then the joint distribution function of *X* = (*x*_1_, *x*_2_, …, *x_n_*) is

(17) $$H\left(X,\varphi_{1},\varphi_{2},\cdots ,\varphi_{n},\theta \right)=C\left(F_{1}\left(x_{1},\varphi_{1}\right),F_{2}\left(x_{2},\varphi_{2}\right),\cdots ,F_{n}\left(x_{n},\varphi_{n}\right),\theta \right),$$

where *C* is the copula function.

The corresponding probability density function is

(18) $$h\left(X,\varphi_{1},\varphi_{2},\cdots ,\varphi_{n},\theta \right)=c\left(F_{1}\left(x_{1},\varphi_{1}\right),F_{2}\left(x_{2},\varphi_{2}\right),\cdots ,F_{n}\left(x_{n},\varphi_{n}\right),\theta \right)\cdot \prod_{i=1}^{n}f_{i}\left(x_{i};\varphi_{i}\right).$$

If the sample is known to be ${\left\{\left(x_{1j},x_{2j},\ldots ,x_{nj}\right)\right\}}_{j=1}^{k}$, then the unknown function’s likelihood function is

(19) $$L\left(\theta \right)=\prod_{j=1}^{k}\left\{c\left(F_{1}\left(x_{1j},\varphi_{1}\right),F_{2}\left(x_{2j},\varphi_{2}\right),\cdots ,F_{n}\left(x_{nj},\varphi_{n}\right),\theta \right)\cdot \prod_{i=1}^{n}f_{i}\left(x_{ij},\varphi_{i}\right)\right\}.$$

The corresponding log-likelihood function is

(20) $$\ln L\left(\theta \right)=\underset{L_{c}}{\underset{︸}{\sum_{j=1}^{k}\ln c\left(F_{1}\left(x_{1j},\varphi_{1}\right),F_{2}\left(x_{2j},\varphi_{2}\right),\cdots ,F_{n}\left(x_{nj},\varphi_{n}\right),\theta \right)}}+\underset{L_{i}}{\underset{︸}{\sum_{j=1}^{k}\sum_{i=1}^{n}\ln f_{i}\left(x_{ij},\varphi_{i}\right)}}.$$

The above method uses the ordinary maximum likelihood estimation method. However, when there are multiple distribution parameters, the calculation becomes more complex. An improved method is to use a two-stage maximum likelihood estimation method. More precisely, the marginal distribution and copula function parameter estimation are used separately, so as to simplify the calculation. The specific algorithm is as follows.

First, the parameter estimation of each marginal distribution function is conducted, and the results are substituted into Equation (21)

(21) $${\hat{\varphi }}_{i}=ArgMaxl_{i}\left(\varphi_{i}\right)=ArgMax\prod_{j=1}^{k}f_{i}\left(x_{ij},\varphi_{i}\right).$$

Then, the maximum likelihood estimation method is used to estimate the parameters of the copula function based on the following equation:

(22) $$\hat{\theta }=ArgMaxl_{c}\left(\theta \right)=ArgMax\sum_{j=1}^{k}\ln c\left(F_{1}\left(x_{1j},{\hat{\varphi }}_{1}\right),F_{2}\left(x_{2j},{\hat{\varphi }}_{2}\right),\cdots ,F_{n}\left(x_{nj},{\hat{\varphi }}_{n}\right),\theta \right).$$

#### 3.2.4. Calculation of the Copula Entropy Value

The copula entropy can be obtained by solving the multiple integrations of Equation (22). However, due to the complexity of the copula function, the form of the integrand function is also very complicated. When this method is used to calculate the copula entropy function, there is a situation where the calculated amount is too large to be calculated. Therefore, this paper uses the Monte Carlo simulation method for the calculation.

The key to the copula entropy calculation is to calculate the integral of the multivariate function using the idea of simulation sampling. Therefore, the main task of this step is to find the area that is completely surrounded by the coordinate surface with the range of *V*_0_. The Monte Carlo method will be used in the surrounded area to sample *N* times (*N* ≥ 10,000). The frequency of the sample points falling into the area of the integrand is then calculated. The percentage of the integral volume and the closed volume based on the frequency is then calculated. Finally, the copula entropy is calculated.

## 4. Case Study

**Figure 2:**
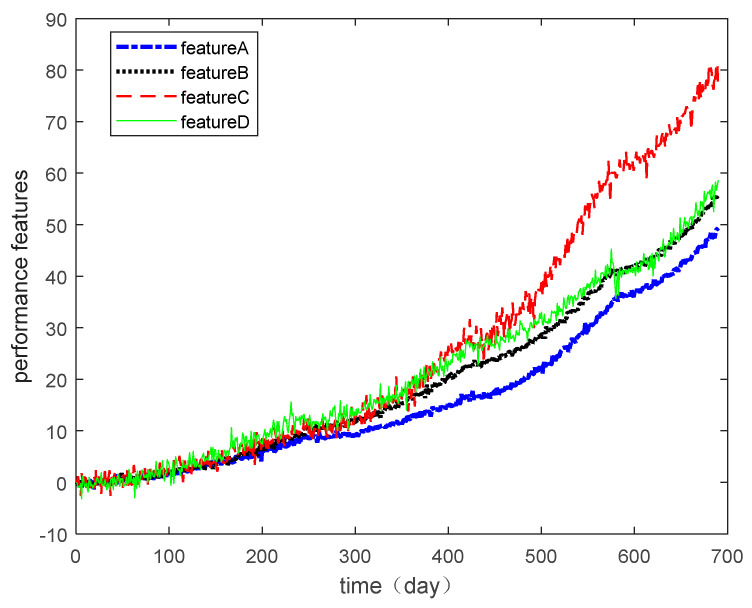
Multi-performance features of degradation data.

**Figure 3:**
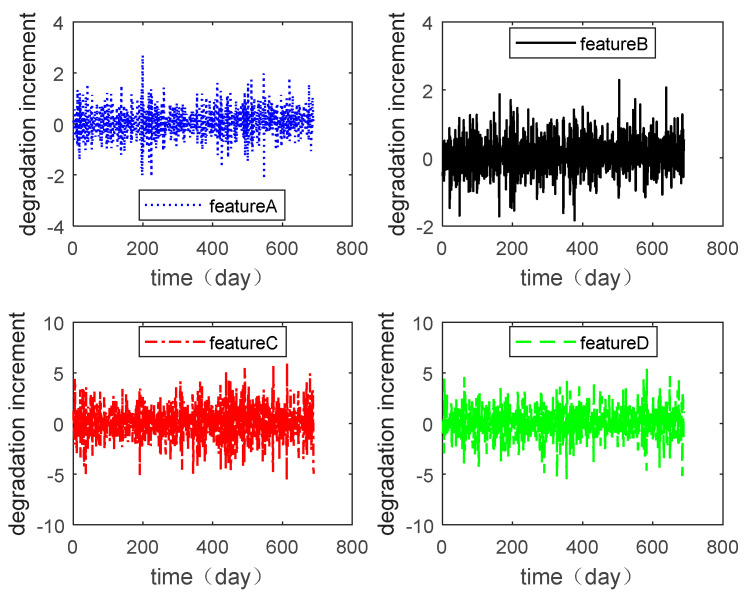
Degradation of various performance features.

In this section, the multiple degradation data of a microwave electronic assembly were used to verify the copula entropy measurement method. To evaluate the lifetime and reliability of the microwave electronic assembly, the degradation data of four performance features collected simultaneously were measured. These performance features were the power gains A, B, C, and D. The degradation data of each performance feature are shown in Figure 2, and the degradation increment data are shown in Figure 3.

Degradation data of the microwave electrical assembly under different operating conditions were used as the validation data in this section. During the same user experience and under certain environmental/operational conditions, the degradation data of the different performance features of the product showed nonlinear dependence relationships due to the complex internal structure of the product. If the dependence among the degradation data is ignored for degradation modeling and lifetime estimation, the result will have lower accuracy. Therefore, to model the degradation more accurately and estimate the service life and reliability of the product effectively, it was necessary to measure dependencies among the degradation features accurately.

### 4.1. Dependence Measurement of Bivariate Degradation Processes

One advantage of copula entropy is that it has dimension. Then the dependence among the variables can be directly compared. Therefore, this section will use the multivariate degradation data of a microwave electrical assembly to verify the copula entropy measurement of the dependence between binary variables.

**Figure 4:**
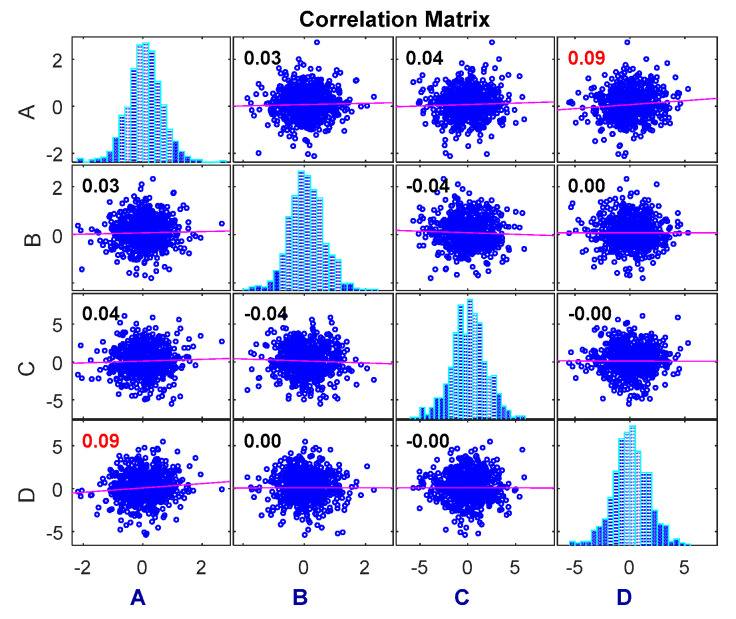
Pearson correlation coefficient measurement.

The dependence of the degradation increment measured using the Pearson correlation coefficient method is shown in Figure 4. This is compared with the experimental results to verify the proposed method.

As can be seen in Figure 4, the correlation coefficient method can only measure the dependence among linear relationships, and the results cannot be compared to obtain different dependencies among the different variables. Therefore, the copula entropy method is proposed to measure the dependence between two sets of data and to compare the dependence between them.

**Figure 5:**
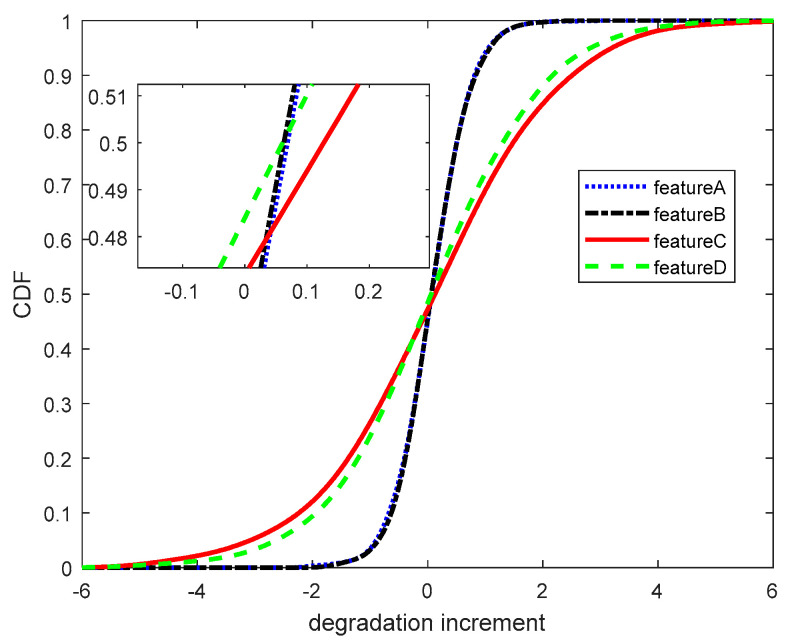
The CDF of degradation increments.

First, the kernel density estimation method was used to calculate the cumulative distribution function (CDF) of each performance feature degradation data increment, as shown in Figure 5.

Second, the Gaussian copula, Frank copula, Clayton copula, and Gumbel copula are used to couple the degradation increase distributions of the different performance features. The AIC criterion is applied to select the most suitable copula function to calculate the copula entropy and quantify the dependence among them. The AIC results are shown in Table 3.

Third, the maximum likelihood estimation method is used to estimate the copula parameters for each of the two degradation increment CDFs. The results are shown in Table 4.

Then, after the copula parameters were determined, the joint PDF of the multivariable was determined. The copula entropy was utilized to quantify the dependence among different features. The binary copula entropy can be calculated as follows:

(23) $$Hc(u,v)=-\int_{0}^{1}\int_{0}^{1}c(u,v)\ln(c(u,v))du,dv.$$

**Figure 6:**
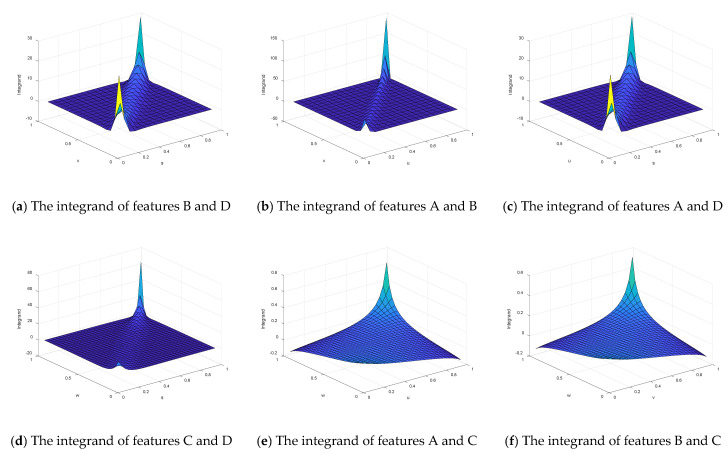
The integrand of different marginal distributions.

Finally, since the form of *c*(*u*, *v*)ln*c*(*u*, *v*) is very complicated, the calculation of the integral will cause computational difficulties. Therefore, the Monte Carlo sampling method was utilized to calculate the copula entropy. Therefore, the integrand in Equation (23) needs to be calculated, namely *c*(*u*, *v*) ln*c*(*u*, *v*). In addition, the Monte Carlo simulation method was used to calculate the copula entropy of each performance feature. According to Equation (23), the integrand functions based on different marginal distributions are shown in Figure 6.

**Figure 7:**
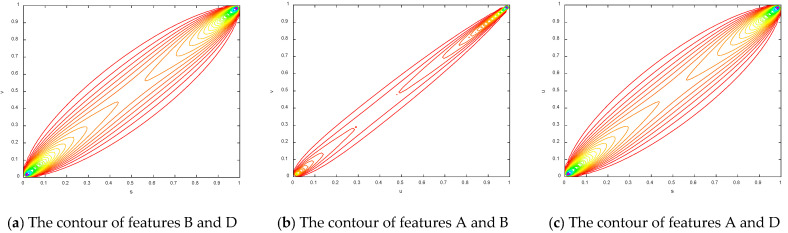

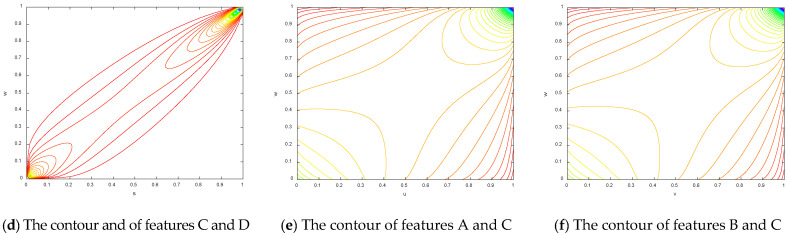
The contours of different copula entropy.

To make the results more obvious, the contours of the copula entropy are shown in Figure 7.

The value of the volume is then calculated depending on the sampling method used. The calculated copula entropy results of each set of performance features are shown in Table 5.

The principle of copula entropy shows that the value of entropy is negatively correlated with the degree of dependence. The dependence of any two variables can then be compared regardless if there exists an intermediate variable among them. In addition, the degree of dependence among variables can be sorted according to the value of the copula entropy among the variables. However, the existing dependence measurement methods fail to do this and cannot compare the degree of dependence without intermediate variables. Therefore, the data in the table show that the dependencies are arranged in descending order: BD, AB, AD, CD, AC, BC.

### 4.2. Dependence Measurement of Different Phases

One advantage of copula entropy is that it can be used to compare the dependencies of multiple feature degradation processes over time, and it can determine the time-varying law of the dependence. The dependence may be variational under different operational conditions. The influence of operational conditions on different degradation features is different. If the operational conditions transform, the degradation rules of different features will change in different ways, which will lead to variation in the dependence among the features.

In this case, the same data used in Section 4.1 were used. It can be seen in Figure 2 that the degradation trend of the variables is not invariable but presents different degradation rates. The dependence among these four sets of degradation data changes with time. However, if only one value that represents the dependence of the variables is obtained, then the degree of dependence among variables at different time phases cannot be compared. Therefore, subsection calculation was needed to compare the variation trend of the dependence of different subsections. Then the dependence among variables can be described more accurately.

**Figure 8:**
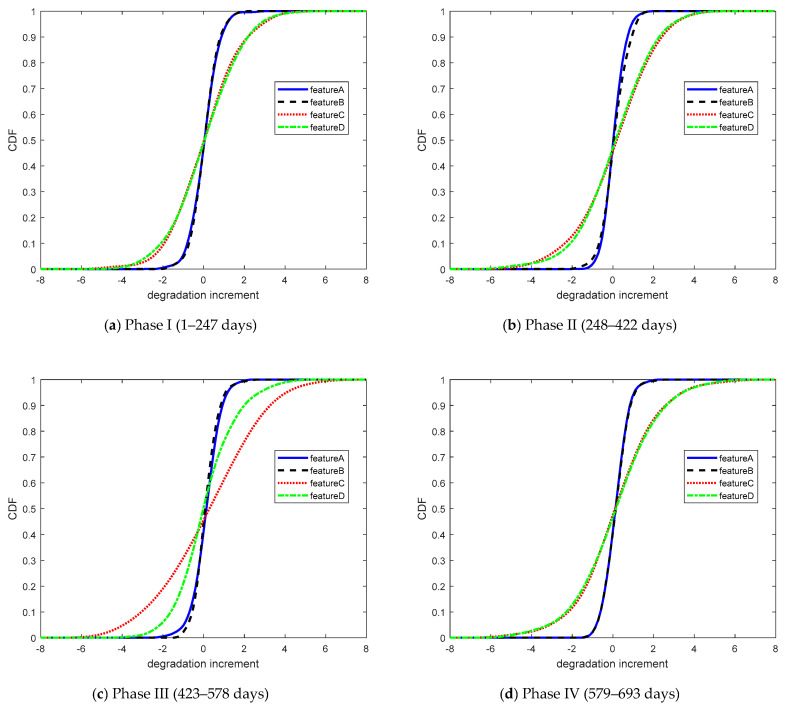
The CDFs of the four sets of degradation data increments.

In this case, the degradation data of performance features A, B, C, and D were divided into four phases in time with 247, 174, 155, and 114 sets of data, respectively. According to Equation (22), the copula entropies of the different stages were calculated to compare the regularity of dependence with time. Copula entropy was used to measure the dependencies for each group of data to analyze the data dependence during different phases. The cumulative distribution function of the four sets of degradation data increments is shown in Figure 8.

Degradation increments were coupled using the Gaussian copula, Frank copula, Clayton copula, and Gumbel copula at each stage, and the AIC for each set of data is shown in Table 6.

The estimation of the copula parameters and the copula entropy results of the total data and the four phrases for the four performance feature degradation increments are shown in Table 7.

When Equation (22) was applied to calculate the value of the copula entropy for the total data in the case, only a value representing the dependence of four variables in this degradation process could be obtained. As shown in Table 7, the value of the copula entropy of the total data among the four variables was −2.7393, and it can be seen that there was dependence among the variables, but it was not significantly strong. In addition, the dependence did not seem to change over time. However as can be observed from the segmentation results in the table, the degree of dependence of the four performance features of the product continuously changed at different phases. The copula entropy of phase IV was the smallest, and the degree of dependence was the highest. Phase I was second, and the next one was phase II. Phase III was the least dependent. Thus, it can be seen that the dependencies of the four performance features changed dynamically with time during the entire degradation process.

### 4.3. Discussion

In the study of traditional theory, the dependence of four variables or multiple variables cannot be compared and analyzed. However, we can compare the dependence among four variables or multiple variables using the copula entropy dependence measurement method. As described in the definition of information theory, entropy is the measurement of disorder [21]. The higher the complexity of the system, the less knowledge we have about information, and the higher the value of entropy.

The conclusion obtained from copula entropy is a measurement of the dependence among the variables. This means that the less we understand the information of the dependence structure, the more disorder there is in the system and the higher the value of the copula entropy. The dependence information among variables becomes more obvious when the dependence becomes stronger and the value of the copula entropy decreases.

According to the data in Table 5 and Table 7, the smaller the copula entropy value, the higher the dependence of variables. Therefore, the degree of the dependence can be directly determined according to the size of the copula entropy values among the variables. Furthermore, the dependence among variables can be analyzed. According to the data in Table 7, the copula entropy values among variables are different during the different phases. More succinctly, the dependence among the variables changes with time. The copula entropy method used to measure the dependence among variables can correctly describe the change in variable dependence during different stages.

An analysis of the above two cases shows that the proposed method can not only compensate for the deficiencies in the Pearson correlation coefficient method but also measure the linear and/or nonlinear dependence of multivariate degradation data. The proposed method can also be used to describe the dependence of data during different phases, suggesting that the dependence changes during different phases.

## 5. Conclusions

Degradation modeling has become an efficient method to evaluate the reliability of long lifespan products. Generally, a product may have multiple degradation features. It is unavoidable that there is dependence among multiple degradation features. The dependence structure may be linear or nonlinear. When ignoring the dependence among multiple degradation features, degradation modeling and lifespan estimation may lead to less credible or erroneous results. Therefore, it is safer to describe and measure dependence accurately and reasonably among multiple degradation features prior to modeling. The Pearson linear correlation coefficient and rank correlation coefficient are often used to measure the dependence between two variables. However, they will misestimate the dependence between two variables when the dependence relationship is nonlinear. Furthermore, they cannot be used to detect dependence when more than two variables are involved. There is no particularly suitable method to measure multiple degradation dependence in the present study. Therefore, we introduce copula entropy, which is used in statistics, to overcome the shortcomings of existing methods in measuring multiple degradation dependence.

In this paper, a measurement method for the dependence among multiple degradation processes based on copula entropy has been proposed for products with multiple performance features. The copula entropy was constructed using the copula function and the information entropy theory. Thus, the copula entropy has the advantages of both of them. It can be applied to measure not only the linear dependence but also the nonlinear dependence. Another advantage of the copula entropy method is that it is not confined to bivariate variables. It is valid to use it to compare dependence among two or more variables based on copula entropy. The copula entropy provides an effective way for us to solve the problem of multiple degradation dependence measurements and solves practical problems in engineering practice. A practical case was used to validate the proposed method and the results proved that the proposed method can effectively improve the accuracy of degradation modeling and life estimation in engineering applications.

Overall, the integration of the copula function and information entropy provides useful insights into dependence measurements in multiple degradation modeling. The effectiveness of the copula entropy method in other fields and a comparison with the traditional copula function fitting effect need to be conducted in the future. In addition, the copula method requires further investigation regarding the influence of different constraints on the fitting results during the process of solving the joint distribution equation.

## Figures and Tables

**Table 1:** Some typical copulas.

Copulas	*C*(*u*_1_, …, *u_n_*)	Parameter
Gaussian	$\Phi_{\theta }\left[\Phi^{-1}\left(u_{1}\right),\Phi^{-1}\left(u_{2}\right),\ldots ,\Phi^{-1}\left(u_{d}\right)\right]{\;}^{1}$	θ∈(−1,1)
Clayton	${\left(\sum_{i=1}^{d}u_{i}^{-\theta }-d+1\right)}^{-1/\theta }$	θ∈(0,∞)
Frank	$-\frac{1}{\theta }\ln\left(1+\frac{\prod_{i=1}^{d}\left[\exp\left(-\theta u_{i}\right)-1\right]}{{\left[\exp\left(-\theta \right)-1\right]}^{d-1}}\right)$	θ∈(−∞,∞)\{0}
Gumbel	$\exp\left\{-{\left[\sum_{i=1}^{d}{\left(-\ln u_{i}\right)}^{\theta }\right]}^{1/\theta }\right\}$	θ∈[1,∞)

^1^ Φ is the standard normal distribution function; Φ*_θ_* is the standard normal distribution function of *d* variables; *u_i_* is the cumulative distribution function of each variable; *θ* is the parameter of the copula function.

**Table 2:** Comparison of copula entropy and the correlation coefficient.

Method	Application Scenarios	Concerns	The Number of Variables	Dimension
Correlation Coefficient	Linear	Degree of dependence	Bivariate	Dimensionless
Copula Entropy	Linear/nonlinear	Structure of dependence	Multivariable	Dimension

**Table 3:** AIC based on different copula functions.

Performance Features	Gaussian	Frank	Clayton	Gumbel
AB	−0.54	−10.27	−211.14	−232.55
AC	−0.47	−2.96	−5.10	−24.42
BC	−8.80	−2.90	−2.15	−24.59
AD	−48.55	−3.24	−12.48	−46.55
BD	−48.68	−3.17	−13.39	−46.76
CD	−11.52	−7.02	−20.83	−30.42

**Table 4:** Copula parameter estimation results.

Marginal Distribution Function	Parameter Estimation	Copula Function
AB	8.3911	Gumbel
AC	1.0412	Gumbel
BC	1.0347	Gumbel
AD	0.9538	Gaussian
BD	0.9532	Gaussian
CD	3.3042	Gumbel

**Table 5:** Copula entropy of binary performance feature.

Marginal Distribution Function	Copula Entropy (nat)	Copula Function
BD	−12.1314	Gaussian
AB	−9.3044	Gumbel
AD	−3.4229	Gaussian
CD	−2.6211	Gumbel
AC	−0.0717	Gumbel
BC	−0.0652	Gumbel

**Table 6:** AIC based on different copula families.

Phase	Gaussian	Frank	Clayton	Gumbel
I	−11.47	−194.59	−6.64	−491.19
II	−162.44	−231.15	−7.61	−425.70
III	−6.36	−330.28	−8.76	−8.40
IV	−14.65	−279.30	−9.11	−476.11

**Table 7:** Parameter estimations and the copula entropy calculation results.

Phase	Total Data	I	II	III	IV
Copula Function	Clayton	Gumbel	Gumbel	Frank	Gumbel
Parameter estimation	5.013325	3.359566	4.034455	25.51778	4.448991
Copula entropy	−2.7393	−88.0933	−81.7226	−0.0411	−123.3709
